# Obstructing Colonic Mass: A Case of Recurrent Endometrial Cancer

**DOI:** 10.1155/2015/593786

**Published:** 2015-06-24

**Authors:** Victor Chedid, Mona Arasoghli, Jana G. Hashash

**Affiliations:** Division of Gastroenterology, Hepatology, and Nutrition, University of Pittsburgh, Pittsburgh, PA 15213, USA

## Abstract

A 71-year-old female with a history of endometrial cancer presented to our hospital with bilateral lower quadrant abdominal pain, which had been worsening over the past two months. The pain was associated with constipation, pencil-thin stools, and a 60 lb weight loss. On physical examination, the patient had suprapubic and left lower quadrant abdominal tenderness. Contrast-enhanced CT scan revealed a 6 cm pelvic mass in the left lower quadrant. It was unclear if this mass was arising from the sigmoid colon or abutting it. A colonoscopy to further investigate the mass was pursued and this revealed a moderate 5 cm long stenosis in the sigmoid colon starting at 15 cm from the anal verge. The stenosis was not ulcerated but had a bluish/purplish hue to it circumferentially. Multiple biopsies were obtained from that area and these revealed architectural changes with mild fibrosis but no malignancy. The mass was further explored with CT-guided fine needle aspiration. The results obtained were positive for cytokeratin-7, CA-125, estrogen receptor protein, and PAX-8 confirming that the mass was endometrial in origin.

## 1. Introduction

Endometrial cancer is the leading cause of genitourinary cancers among women in the United States [1]. In general, if diagnosed early, it has a favorable prognosis. The five-year survival for stage I disease is approximately 80 to 90 percent, for stage II it is 70 to 80 percent, and for stages III and IV it is 20 to 60 percent [2]. Recurrence rates are relatively low, with the majority of cases recurring within three years after treatment. Around 77% of recurrences are associated with symptoms related to the site of recurrence [3]. Recurrence sites are evenly distributed between vaginal vault and distant metastasis (lung or abdominal) [3]. Signs or symptoms suggestive of recurrence include vaginal bleeding, abdominal or pelvic pain, persistent cough, or unexplained weight loss. Hence, in females with a history of gynecological cancers, including endometrial cancer, it is important to maintain an index of suspicion for recurrence when they present with such symptoms [3, 4].

## 2. Case Report

A 71-year-old Caucasian woman, with a remote history of stage 1 endometrial cancer treated more than 10 years ago with total abdominal hysterectomy and bilateral oophorectomy (TAHBSO) with no radiation, presented to our institution with intermittent cramping and bilateral lower quadrant abdominal pain, progressively worsening over a 2-month period. This was associated with constipation and thin stools. She also reported a 60 lb weight loss over the past year. She denied any nausea, vomiting, hematochezia, or melena. She also denied fevers, chills, or night sweats. Physical examination revealed a tender abdomen mainly over the suprapubic area and the left lower quadrant. No guarding or rebound tenderness was appreciated. Bowel sounds were normoactive.

**Figure 1:**
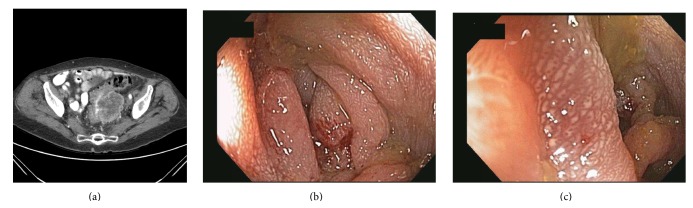
(a) CT scan revealing a 6 cm pelvic mass. (b) Colonoscopy revealing a 5 cm long stenosis in the sigmoid colon starting at 15 cm from the anal verge. (c) Colonoscopy with closer view of stenosis, which was not ulcerated but had a bluish/purplish hue to it.

A CT scan was performed and showed a 6 cm pelvic mass in the left lower quadrant (Figure 1(a)). It was unclear if this mass was arising from the sigmoid colon or abutting it. A colonoscopy was pursued to further evaluate the patient's symptoms and investigate the radiographic findings. There was a moderate stenotic area measuring 5 cm in length in the sigmoid colon starting at 15 cm from the anal verge. This area was easily traversed with an adult colonoscope. The stenosis was not ulcerated but had a bluish/purplish hue to it circumferentially (Figures 1(b) and 1(c)) suspicious for malignant infiltration or extrinsic compression. Multiple biopsies were obtained from that area and these revealed architectural changes with mild fibrosis but no malignancy. The patient subsequently underwent a CT-guided fine needle aspiration of this 6 cm pelvic mass. The cytology results were positive for cytokeratin-7, CA-125, estrogen receptor protein, and PAX-8, confirming that this mass was indeed endometrial in origin. This pelvis mass was abutting the sigmoid colon causing external compression as characterized by the endoscopic stricture and discoloration described above.

Surgical resection of the pelvic mass was performed, followed by chemotherapy and pelvic radiation. The patient did not tolerate the chemotherapy and passed away from complications related to neutropenia and sepsis two months after presentation.

## 3. Discussion

Endometrial cancer is the seventh leading cause of cancer amongst women in the United States with approximately 42,160 new cases diagnosed every year and accounting for 7780 deaths as per 2009 cancer statistics [1]. Ninety percent of endometrial cancer cases are of the endometrioid type, which tend to present at an early stage of disease and are amenable to cure by surgery. The remaining cases (less than 10%) are mostly composed of uterine papillary serous carcinoma and clear cell carcinoma, which constitute almost half of all recurrences. In addition to the histologic type of endometrial cancer, the initial stage at diagnosis is an important predictor of relapse because the more advanced the stage, the higher the risk of micrometastasis [5]. Additional predictors of endometrial cancer recurrence are depth of myometrial invasion, presence of lymph node metastasis, and the presence of extrauterine disease [3]. Endometrial cancer following primary surgery commonly recurs locally in the vaginal vault. It also may recur in the para-aortic and pelvic lymph nodes or at distant sites such as the peritoneum and lungs. Atypical sites include the extrapelvic lymph nodes, liver, adrenals, brain, bone and very rarely the pancreas, spleen, skeletal muscles, and the vulva [6]. High-risk patients at time of diagnosis may receive postoperative adjuvant radiation therapy in the form of vaginal vault brachytherapy, pelvic external-beam radiation therapy, or other modalities [3].

Our patient had a recurrence more than 10 years from diagnosis of endometrial cancer. Her presentation was interesting since she presented with symptoms of bowel obstruction, and her colonoscopy showed evidence of an obstructing colonic mass, which was thought to be primary colon cancer versus metastatic endometrial cancer in the colon wall. While most relapses occur within 3 years after hysterectomy for endometrioid carcinoma, vaginal recurrence 17 years after TAHBSO and adjuvant pelvic external-beam radiation therapy (EBRT) has been reported [7]. Colonic obstruction secondary to recurrent gynecologic malignancies is commonly a consequence of external compression. Palliative stents have been found effective in alleviating symptoms in these patients as compared to surgery [7]. There has been a case report of a patient with endometrial stromal sarcoma infiltrating the ascending colon 7 years after surgery, chemotherapy, and stem cell transplantation [8], but no reports of the endometrioid type. Bowel obstruction 34 years after hysterectomy has been reported, which was found to be secondary to endometrial adenocarcinoma [9].
